# Mogroside V and mogrol: unveiling the neuroprotective and metabolic regulatory roles of *Siraitia grosvenorii* in Parkinson’s disease

**DOI:** 10.3389/fphar.2024.1413520

**Published:** 2024-07-23

**Authors:** Quan Tang, Rui Qiu, Mei Guo, Lili Wang, Yan Zhang, Yuewen Chen, Yong Cheng

**Affiliations:** ^1^ Laboratory of Mass Spectrometry Imaging and Metabolomics, Center on Translational Neuroscience, College of Life and Environmental Sciences, Minzu University of China, Beijing, China; ^2^ Institute of National Security, Minzu University of China, Beijing, China; ^3^ Key Laboratory of Ethnomedicine of Ministry of Education, School of Pharmacy, Minzu University of China, Beijing, China; ^4^ Chinese Academy of Sciences Key Laboratory of Brain Connectome and Manipulation, Shenzhen Key Laboratory of Translational Research for Brain Diseases, The Brain Cognition and Brain Disease Institute, Shenzhen Institute of Advanced Technology, Chinese Academy of Sciences, Shenzhen–Hong Kong Institute of Brain Science—Shenzhen Fundamental Research Institutions, Shenzhen, China; ^5^ Guangdong Provincial Key Laboratory of Brain Science, Disease and Drug Development, HKUST Shenzhen Research Institute, Shenzhen, China

**Keywords:** mogroside V, mogrol, Parkinson’s disease, metabolism, neuroprotection

## Abstract

**Introduction:**

*Siraitia grosvenorii* (Swingle) C. Jeffrey, is an edible and traditional medicine widely used in China. Mogroside V (MGV) and mogrol (MG) are its main active ingredients, which have been found to be effective in the treatment of neurodegenerative diseases recently. However, whether they can effectively treat Parkinson’s disease (PD) and their underlying mechanisms have not been sufficiently explored. In this study, we investigated the neuroprotective and metabolic regulatory effects of MGV and MG on PD.

**Materials and methods:**

Using SH-SY5Y cell models and an MPTP-induced mouse model of PD, we evaluated the compounds’ efficacy in mitigating MPP+-induced neurotoxicity and ameliorating motor deficits and dopaminergic neuron loss. Employing widely targeted metabolomics and bioinformatics analysis to investigate the Metabolic imbalance rectification caused by MGV and MG treatment. The vivo experimental protocol encompassed a 14-day drug administration regimen with mice randomly allocated into six groups (n = 9) receiving distinct compound dosages including a control group, a model group, MGV-H (30 mg/kg/day), MGV-L (10 mg/kg/day), MG-H (15 mg/kg/day), and MG-L (3 mg/kg/day).

**Results:**

Our findings revealed that pre-treatment with MGV and MG significantly enhanced cell viability in SH-SY5Y cells exposed to MPP+, demonstrating a potent protective effect against neurotoxicity. In the MPTP mouse model, MGV-H, MGV-L, and MG-H significantly enhanced motor coordination as assessed by the rotarod test (*p* < 0.05); MGV-L and MG-H evidently inhibited dopaminergic neuronal loss in the substantia nigra pars compacta (*p* < 0.05). Furthermore, metabolomic analysis of the substantia nigra highlighted the restoration of metabolic balance, with MGV-L and MG-H impacting 160 differential metabolites and modulating key pathways disrupted in PD, including sphingolipid metabolism, fatty acid metabolism, and amino acid metabolism. Notably, treatment with MGV-L and MG-H led to the regulation of 106 metabolites, showing a recovery trend towards normal levels, which constitutes approximately 17.5% of the identified metabolites. Key metabolites such as n-acetyl-l-glutamate, hexadecanoic acid, and 9-octadecenal were significantly altered (*p* < 0.05), underscoring their broad-spectrum metabolic regulatory capacity.

**Conclusion:**

This study underscores the potential of natural compounds in developing comprehensive treatment strategies for neurodegenerative diseases, paving the way for future clinical research to validate the therapeutic efficacy of mogrosides in PD.

## 1 Introduction

*Siraitia grosvenorii* (Swingle) C. Jeffrey ex A. M. Lu et Z. Y. Zhang, belonging to the Cucurbitaceae family, is distinguished as a remarkable instance of the “medicine food homology” concept in China (Duan et al., 2023). Native to China’s southern locales, especially notable in Guilin, Guangxi Province, this perennial vine, commonly referred to as Luo Han Guo, holds a cherished position in traditional medicine (Lu and Zhang, 1984; Wu et al., 2022). The dried fruits of Luo Han Guo are traditionally used for their therapeutic properties, such as dissipating heat, alleviating coughs, moisturizing the lungs and intestines, and facilitating bowel movements (Li et al., 2014; Soejarto et al., 2019; Lin et al., 2023). Among its bioactive constituents, MGV stands out for its abundance and efficacy. Additionally, MG, the aglycone form of mogrosides, is produced through the metabolism of MGV, involving the detachment of five glucosides in the digestive system, thereby enabling MG to cross the blood-brain barrier and distribute within the brain (Xu et al., 2015). The neuroprotective properties of MGV and MG have been acknowledged (Chen et al., 2019; Ju et al., 2020; Luo et al., 2022; Liu et al., 2023), prompting further investigation into their potential benefits.

Parkinson’s disease (PD) emerges as a widespread neurodegenerative condition predominantly affecting the elderly, with more than 2% of individuals over the age of 65 worldwide being afflicted (Poewe et al., 2017). PD is characterized by a spectrum of motor symptoms, including bradykinesia, muscle rigidity, resting tremor, and postural instability, alongside non-motor symptoms such as sleep disturbances, anxiety, depression, cognitive deficits, and constipation (Kalia and Lang, 2015; Bloem et al., 2021). The disease’s neuropathological signature is marked by the degeneration and loss of dopaminergic neurons in the substantia nigra (SN) and the abnormal aggregation of α-synuclein within cells (Armstrong and Okun, 2020; Bloem et al., 2021). While oxidative stress and inflammation mediated by cytokines have long been focal points in PD research (Chen et al., 2018; Wei et al., 2018), recent advancements in metabolomics have revealed metabolic alterations in both PD patients and animal models, particularly noting dysregulation in the striatum and SN (Lu et al., 2018; Zhang et al., 2023).

Given the potential neuroprotective effects of MGV and MG, a primary aim of this study is to explore their therapeutic potential in the context of PD. This exploration is particularly timely, as current treatments for PD are largely symptomatic and do not halt disease progression. Understanding the metabolic underpinnings of PD and how MGV and MG might influence these pathways could pave the way for novel therapeutic strategies. To achieve this, our research conducts a comprehensive metabonomic analysis of the SN from mice treated with MGV and MG. This investigation into the metabolic profile alterations seeks to uncover the mechanisms by which MGV and MG exert their neuroprotective effects, potentially influencing the complex molecular pathways involved in PD. By elucidating these mechanisms, our study not only aims to deepen the scientific community’s understanding of the neuroprotective actions of MGV and MG but also to investigate their viability as therapeutic agents against PD. Through this research, we endeavor to expand the treatment landscape for PD, offering new hope and possibilities for those facing this debilitating disease.

## 2 Material and methods

### 2.1 Materials and reagents

Mogroside V (CAS:88901-364) and Mogrol (CAS: 88930-15-8) were purchased from Chengdu Lemaitian Pharmaceutical Technology Co., Ltd. (Chengdu, China). MPTP and MPP+ were purchased from Macklin (Shanghai, China). MTT assay kit was given by Solarbio (Shanghai, China). Antibodies against Tyrosine hydroxylase (TH), β-actin were recruited from Cell Signaling Technology (Shanghai, China). Secondary antibodies were recruited from ZSGB-Bio (Beijing, China).

### 2.2 Cell culture and treatment

Human neuroblastoma SH-SY5Y cells were obtained from American Type Culture Collection (ATCC, Manassas, VA, United States) and cultured in DMEM containing 10% fetal bovine serum, 1% stable glutamine, and antibiotics, under a 5% CO2 atmosphere at 37°C. The SH-SY5Y cells were seeded at a density of 5 × 10 ^ 4 cells/mL in a 96-well plate and incubated for 24 h until reaching stable growth. Subsequently, the cells were subjected to MPP+ damage for 20 h, and their viability was assessed using the MTT assay. A gradient of MPP+ concentrations (1, 2, 3, 4, 5 mM) was employed to determine the optimal damaging concentration. Following a 24-h incubation of SY5Y cells, MPP+ and varying doses of MGV (10, 50, 100 μM) and MG (10, 50, 100 μM) were administered to evaluate their neuroprotective effects.

### 2.3 Animals and treatment

Eight-week-old male C57BL/6J mice (20 ± 2 g) were obtained from Vital River Laboratory (Beijing, China). After a week of acclimatization, the animals were housed under controlled conditions (24°C ± 1°C, 50% ± 1% humidity, and a 12 h light/dark cycle) with *ad libitum* access to food and water. All behavioral experiments were conducted in accordance with the guidelines approved by the Animal Care and Use Committee of Minzu University of China (Approval No. ECMUC2019001AO).

**FIGURE 1:**
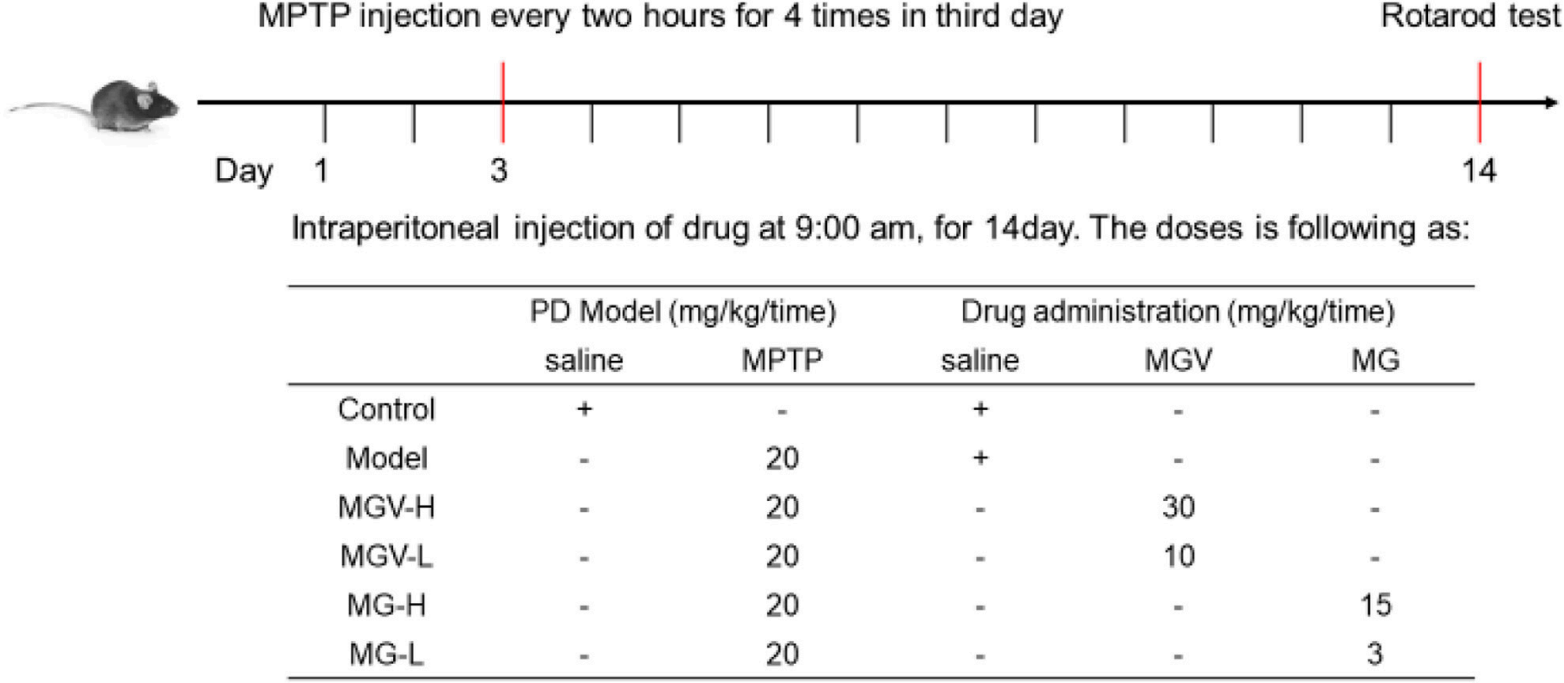
The vitro experimental design.

The drug administration procedures are depicted in Figure 1 and span a period of 2 weeks. To establish the Parkinson’s disease (PD) model, mice were intraperitoneally injected with 1-Methyl-4-phenyl-1,2,3,6-tetrahydropyridine (MPTP) (Macklin, Shanghai, China) on the third day. Mice were then randomly assigned to six groups (n = 9 each): Control, Model, MGV-H, MGV-L, MG-H, and MG-L. In detail, Control: saline injections; Model: Mice received MPTP and saline injections; MGV-H (high dose of MGV): Mice received MPTP and MGV (30 mg/kg/day); MGV-L (low dose of MGV): Mice received MPTP and MGV (10 mg/kg/day); MG-H (high-dose MG): Mice received MPTP and MG (15 mg/kg/day); MG-L (low dose of MG): Mice received MPTP and MG (3 mg/kg/day).

### 2.4 Behavioral analysis: rotarod test

Motor coordination and balance were evaluated using the rotarod test on the final day of the experiment, as previously described (Sun et al., 2020). Briefly, mice underwent 3 days of pretraining at 10 rpm until they could stay on the rod for a minimum of 90 s. During the test, the rod’s speed was increased from 4 to 40 rpm over 5 min. The time each mouse remained on the rod before falling was recorded.

### 2.5 Brain immunohistochemistry

Subsequent to the rotarod test, the mice were euthanized with pentobarbital and perfused with saline. The brains were fixed in 4% paraformaldehyde, cryoprotected in sucrose solutions, sectioned at 40 μm using a cryostat, and stored in a cryoprotectant at −20°C. Following washing with PBS and treatment with 3% H2O2, the brain sections were blocked with 10% goat serum and 0.3% Triton X-100 and then incubated with primary antibodies against TH (CST, Boston, MA, United States) at 4°C, followed by incubation with secondary antibodies (goat anti-rabbit, 1:400) at room temperature (approximately 25°C). The sections were mounted, dehydrated, and cover-slipped prior to bright-field microscopy analysis.

### 2.6 Neuron counting

The half brain slices of the SN compacta were obtained by freezing and slicing the brain into continuous 40 μm-thick slices. Every fourth slice underwent immunohistochemical staining, and TH positive neurons in the SN were manually counted under a light microscope. The neuron count from one slice was multiplied by four to represent the number of TH positive neurons in half of the brain’s SN, and then further multiplied by two to estimate the total number in the whole brain’s SN. Subsequently, this count was utilized for the statistical analysis of neurons in the SN compacta.

### 2.7 Western blot analysis

The SN and striatum were dissected, homogenized in RIPA buffer, and analyzed for protein concentration using a BCA Protein Assay Kit. Samples were prepared with 4× loading buffer, denatured at 95°C for 5 min, and subjected to SDS‒PAGE. The proteins were then transferred to a nitrocellulose membrane, blocked with 5% skim milk, and incubated with primary and secondary antibodies, after which the proteins were visualized via enhanced chemiluminescence.

### 2.8 Metabolite measurement

Targeted metabolomics of SN samples from the Control, Model, MGV-L, and MG-H groups was conducted using UPLC‒MS/MS (Shim-pack UFLC SHIMADZU CBM30A system; 4500 QTRAP, Applied Biosystems). Metabolites were identified using the MetWare database and quantified via multiple reaction monitoring.

### 2.9 Differential expression analysis

Orthogonal projections to latent structures discriminant analysis (OPLS-DA) was applied to assess the differential expression of metabolites. Variable importance in projection (VIP) values were extracted from the model. Metabolites with VIP ≥ 1 and an absolute Log_2_FC (fold change) ≥ 2, as determined by the Mann‒Whitney U test, were considered to be differentially expressed (Du et al., 2021).

### 2.10 Bioinformatics analysis

Differentially expressed metabolites were annotated using the Kyoto Encyclopedia of Genes and Genomes (KEGG) and analyzed for pathway enrichment with MetaboAnalyst software as described in the previously published literature (Du et al., 2023). The enrichment significance was set at *p* < 0.05. Standardizing and centering the relative content of differentially abundant metabolites, followed by K-means clustering analysis, was used to study the relative variation trends of metabolites in different samples.

### 2.11 Statistical analysis

Statistical analysis was performed using GraphPad Prism 8.0. The data are presented as the mean ± SEM. Student’s t-test assessed group differences, while one-way ANOVA and multiple test comparisons analyzed differences between independent groups. *p* < 0.05 indicates a statistically significant difference.

## 3 Results

### 3.1 Protective effects of MGV and MG against MPP+-induced neurotoxicity in SH-SY5Y cells

**FIGURE 2:**
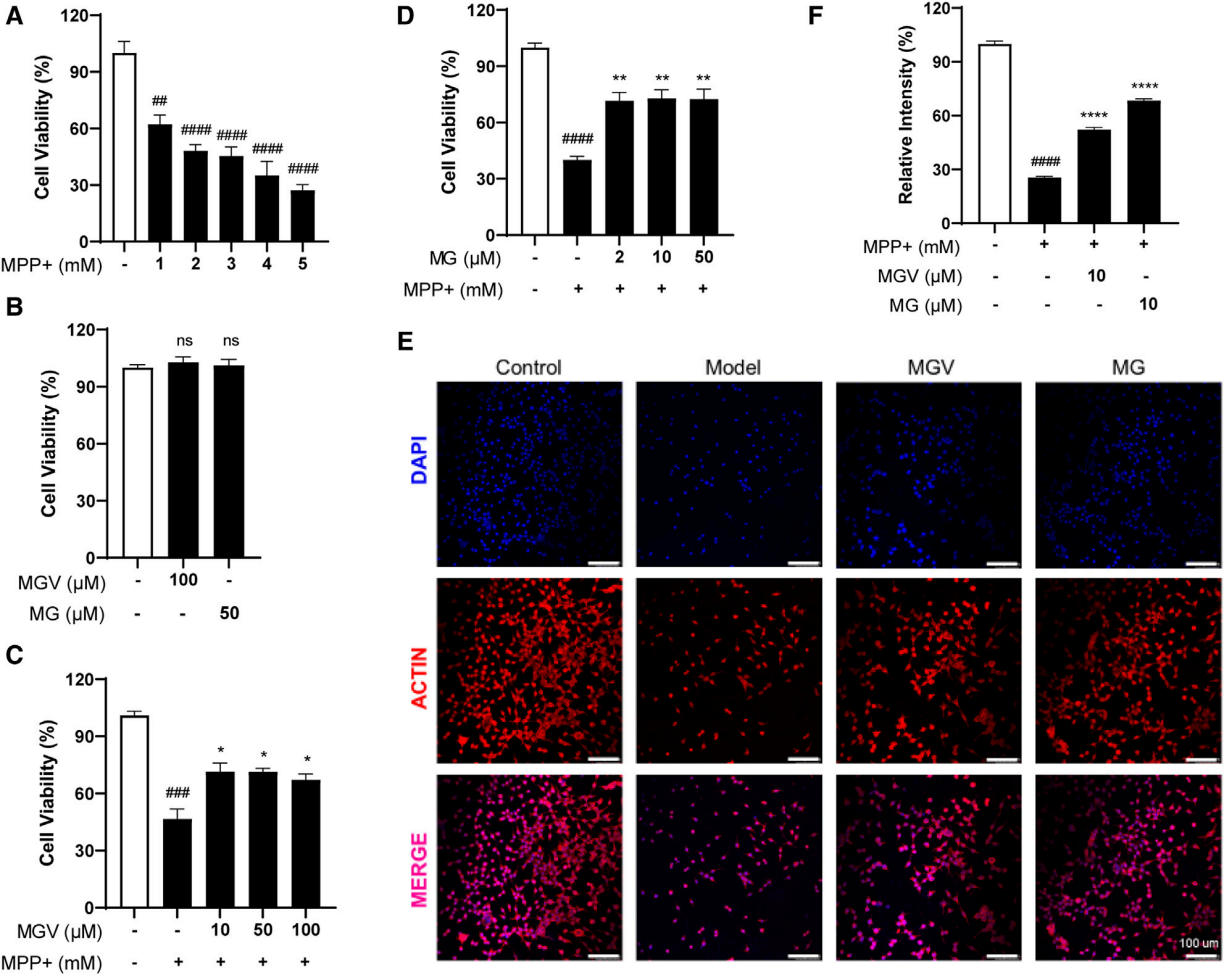
Mogroside V and Mogrol attenuate MPP**+**-induced Cell Damage in SH-SY5Y Cells. **(A)** Establishment of PD cell model; **(B)** 100 uM MGV and 50 uM MG are not toxic to SH-SY5Y cells; **(C)** MGV and **(D)** Mogrol alleviated damage in PD cell model; **(E)** Actin immunofluorescence staining images (Scale bar: 100 μM) and **(F)** quantitative analysis of actin level in SH-SY5Y cells. Data were expressed as mean ± SEM. ns, #*p* < 0.05, ##*p* < 0.01, ###*p* < 0.001, vs. Control group; *p < 0.05, **p < 0.01, ***p < 0.001, vs. Model group; ns, no significance.

The SH-SY5Y cell line, a dopaminergic neuronal model, is widely used in Parkinson’s disease (PD) research to study neurotoxicity and neuroprotection (Xie et al., 2010). In this study, MPP+, a neurotoxin, was utilized to induce a PD-like condition by causing cellular toxicity (Xicoy et al., 2017). Initial experiments evaluated the protective capabilities of MGV and MG against MPP+-triggered neurotoxicity in SH-SY5Y cells. Exposure to MPP+ at varying concentrations (1–5 mM) significantly reduced cell viability, with a concentration of 2 mM notably halving cell survival (Figure 2A). Compared to the control group, the introduction of 100 uM MGV and 50 uM MG did not show any significant impact on cell viability (Figure 2B), suggesting that concentrations equal to or below this threshold are non-toxic to the cells. Strikingly, pre-treatment with either MGV (10, 50, 100 μM) or MG (10, 50, 100 μM) effectively mitigated MPP+-induced cell death, as evidenced in Figures 2C, D. Notably, the average cell viability levels and statistical significance were the highest with 10 μM MGV (71.59 ± 4.421, *p* < 0.05) and 10 μM MG (72.80 ± 4.602, *p* < 0.01). Consequently, we selected 10 μM MGV and 10 μM MG for actin immunofluorescence staining. As depicted in Figures 2E, F, the results demonstrated a significant decrease in cell count and neurite branching following MPP+ treatment, effects that were effectively attenuated by pretreatment with MGV (*p* < 0.05) or MG (*p* < 0.05). These results confirm the potent protective effect of MGV and MG against MPP+-induced damage in SH-SY5Y cells, underscoring their potential as neuroprotective agents in PD research.

### 3.2 Alleviation of MPTP-induced motor dysfunction and neuronal degeneration by MGV and MG

**FIGURE 3:**
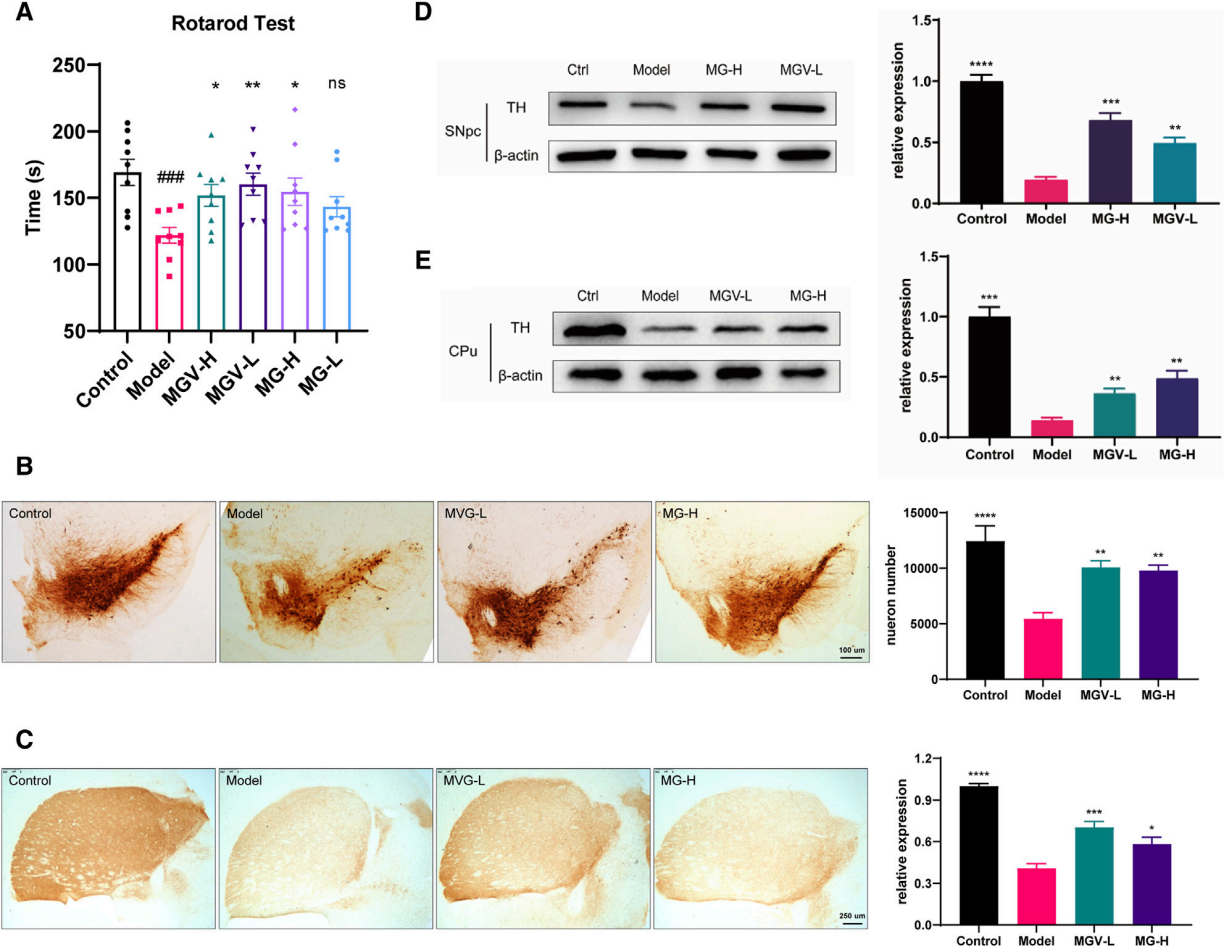
Mogrosides ameliorates MPTP-induced Motor dysfunction and Attenuates MPTP-Induced Loss of Dopaminergic Neurons in the SN and Striatum. **(A)** The exercise time of different groups of mice in the rotarod test after drug treatment. Th immunofluorescence staining images and statistical results of **(B)** substantia nigra and **(C)** striatum in different groups. Statistical results of TH and Actin protein content and TH/actin relative content in **(D)** substantia nigra and **(E)** striatum of different groups. Data were expressed as mean ± SEM. #*p* < 0.05, ##*p* < 0.01, ###*p* < 0.001, vs. Control group; *p < 0.05, **p < 0.01, ***p < 0.001, vs. Model group; ns, no significance.

To further investigate the neuroprotective effects of MGV and MG, an MPTP-induced PD mouse model was developed as previously reported (Sun et al., 2023), mimicking PD-like symptoms. Motor function, assessed using the rotarod test on day 14, showed severe impairments in MPTP-treated mice compared to controls. Notably, both MGV and MG-H significantly enhanced motor performance in MPTP-treated mice, whereas a low dose of MG showed no effect, suggesting a dose-dependent response (Figure 3A).

The degeneration of dopaminergic neurons in the substantia nigra pars compacta (SNpc) represents a critical pathological aspect of PD. TH, essential for dopamine synthesis, is used as a marker for dopaminergic neurons. Analysis revealed a profound reduction in TH-positive neuron density within the SNpc and caudate putamen (CPu) in the MPTP model group compared to controls. However, both MGV-L and MG-H treatments significantly preserved TH-positive neurons in these areas (Figures 3B, C). Western blot analysis supported these findings, showing a significant decrease in TH expression in MPTP-treated groups, which was effectively reversed by treatments with MGV-L or MG-H in both the SNpc and CPu (Figures 3D, E). These combined results highlight the substantial neuroprotective capabilities of MGV-L and MG-H, offering promising avenues for addressing dopaminergic neuronal loss and motor dysfunctions associated with PD.

### 3.3 Metabolic reconstitution by MGV and MG in a Parkinson’s disease mouse model

**FIGURE 4:**
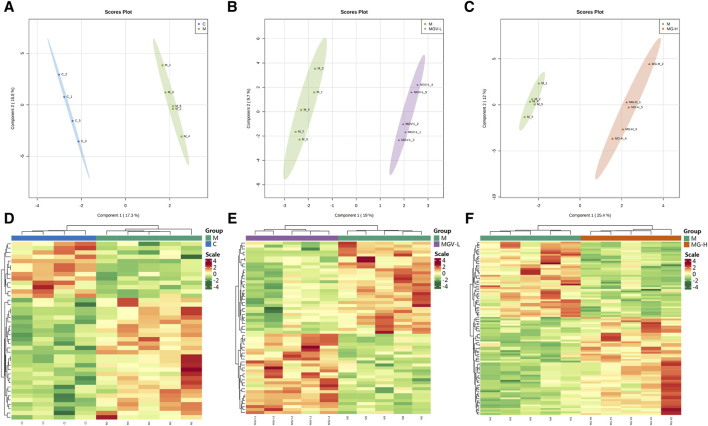
Differential expression of metabolites between the model group and other groups. After PCA analysis, OPLS-DA scores of model group compared with **(A)** C, **(B)** MGV, **(C)** MG. Heatmap analysis of differential metabolites of **(D)** C, **(E)** MGV, **(F)** MG compared with the model group.

We next explore the impact of MGV and MG on metabolic alterations within the SN of a Parkinson’s disease (PD) mouse model, employing widely targeted metabolomics to delineate the metabolic changes and pathways influenced by these compounds. A comprehensive analysis identified 605 metabolites across four groups: Control (C), Model (M), MGV-L and MG-H. Principal component analysis (PCA) compared the C, MGV-L, and MG-H groups against the Model group, revealing significant principal component variances indicative of metabolic differences. The OPLS-DA (Figures 4A–C) and subsequent model validation (Supplementary Figure S1) underscored these distinctions.

**FIGURE 5:**
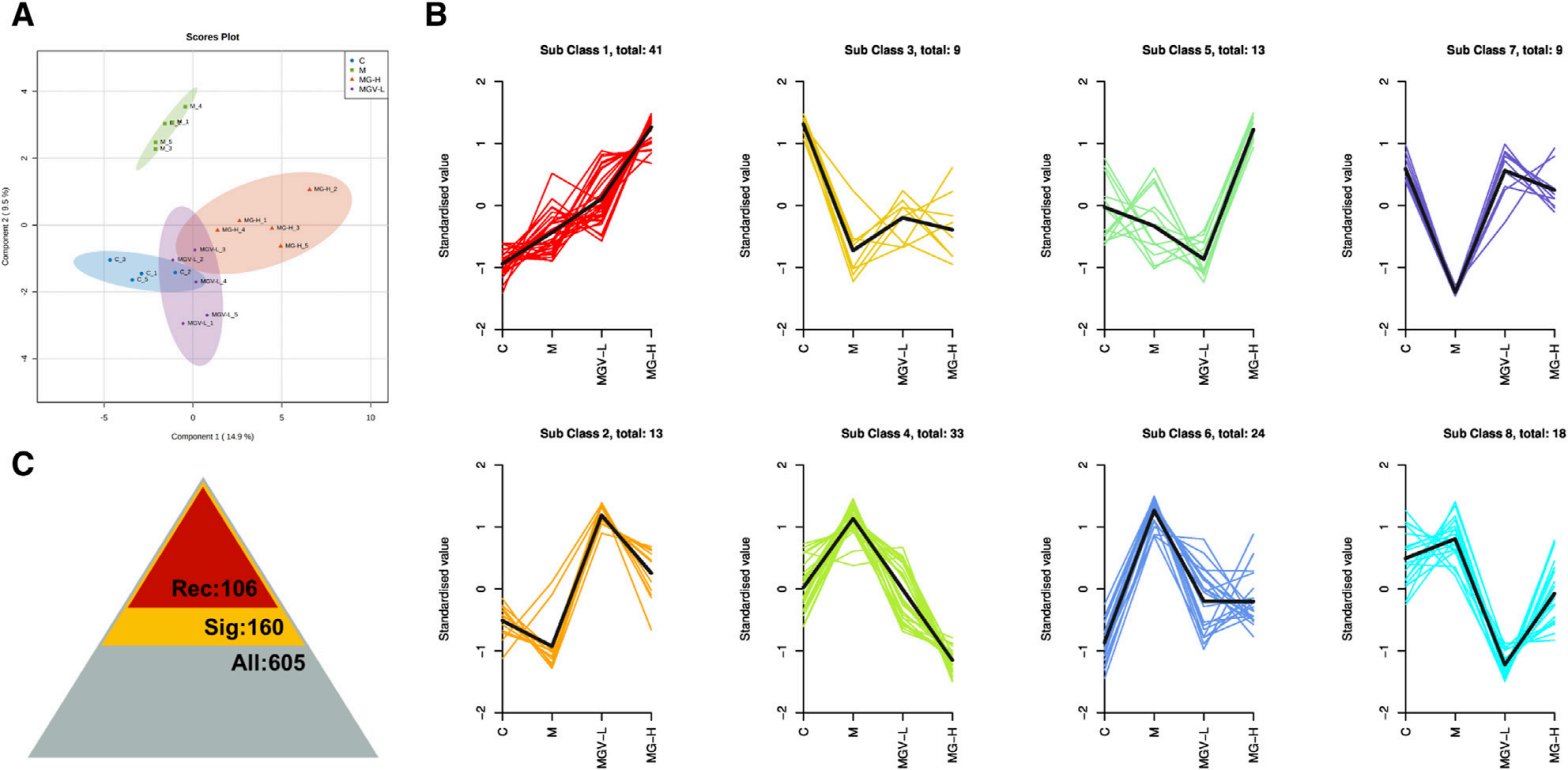
Mogroside V and Mogrol treatment restored metabolism in PD mice. **(A)** OPLS-DA score chart, **(B)** K-means value analysis result chart, **(C)** statistical chart of significance analysis of all metabolites, the number of identified metabolites is 605 (gray), the number of differential metabolites is 160 (yellow), and the number of metabolites showing recovery trend after administration is 106 (red).

**FIGURE 6:**
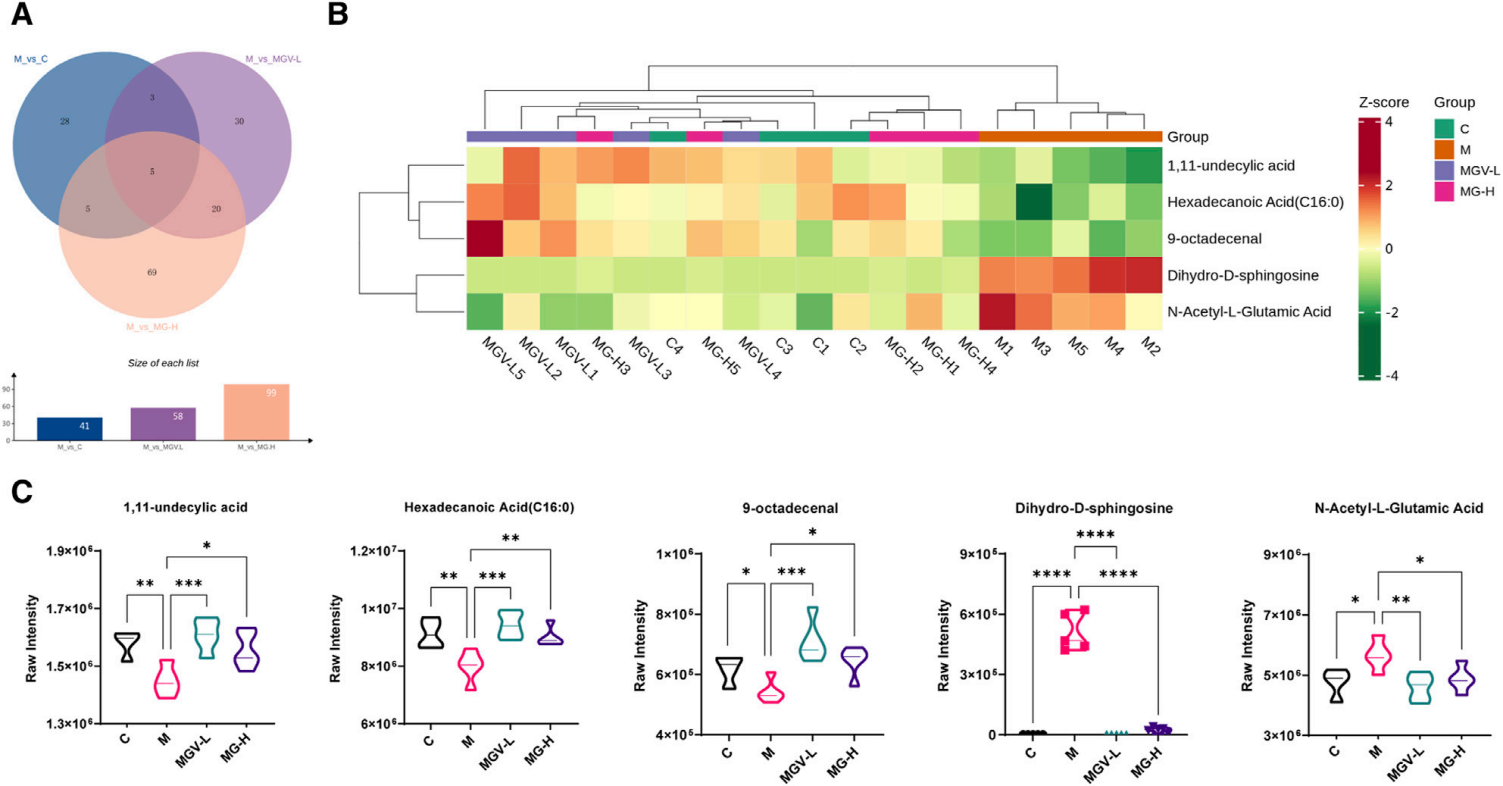
Analysis result of differential metabolites. **(A)** Venn diagram showed that five key differential metabolites were present in all three groups; **(B)** Heatmap analysis of key differential metabolites; **(C)** violin diagram of single key differential metabolites. *p < 0.05, **p < 0.01, ***p < 0.001, vs. Model group.

Differential metabolite expression was quantified through a combination of VIP scores from OPLS-DA and Mann-Whitney U test *p*-values. This approach identified 61, 65, and 122 differentially expressed metabolites in the C, MGV-L, and MG-H groups, respectively, compared to the Model group (Supplementary Tables S1–S3). Clustering heat maps visualized the regulation patterns of these metabolites, showing notable upregulation and downregulation trends across the comparisons (Figures 4D–F). Further PCA of the entire dataset revealed a proximal distribution of MGV-L, MG-H, and C groups, contrasting with the Model group’s distinct separation (Figure 5A). K-mean analysis of 160 differential metabolites highlighted a recovery trend in approximately 17.5% of the identified metabolites following treatment with MGV and MG (Figures 5B, C). Key metabolites bridging the control and treatment groups included 1,11-undecylic acid, hexadecanoic acid (c16:0), 9-octadecenal, dihydro-d-sphingosine, and n-acetyl-l-glutamic acid (Figure 6A). Their expression patterns and significant alterations were depicted through a clustering heat map (Figure 6B) and violin plots (Figure 6C), showcasing the regulatory effects of mogrosides on these metabolites.

**FIGURE 7:**
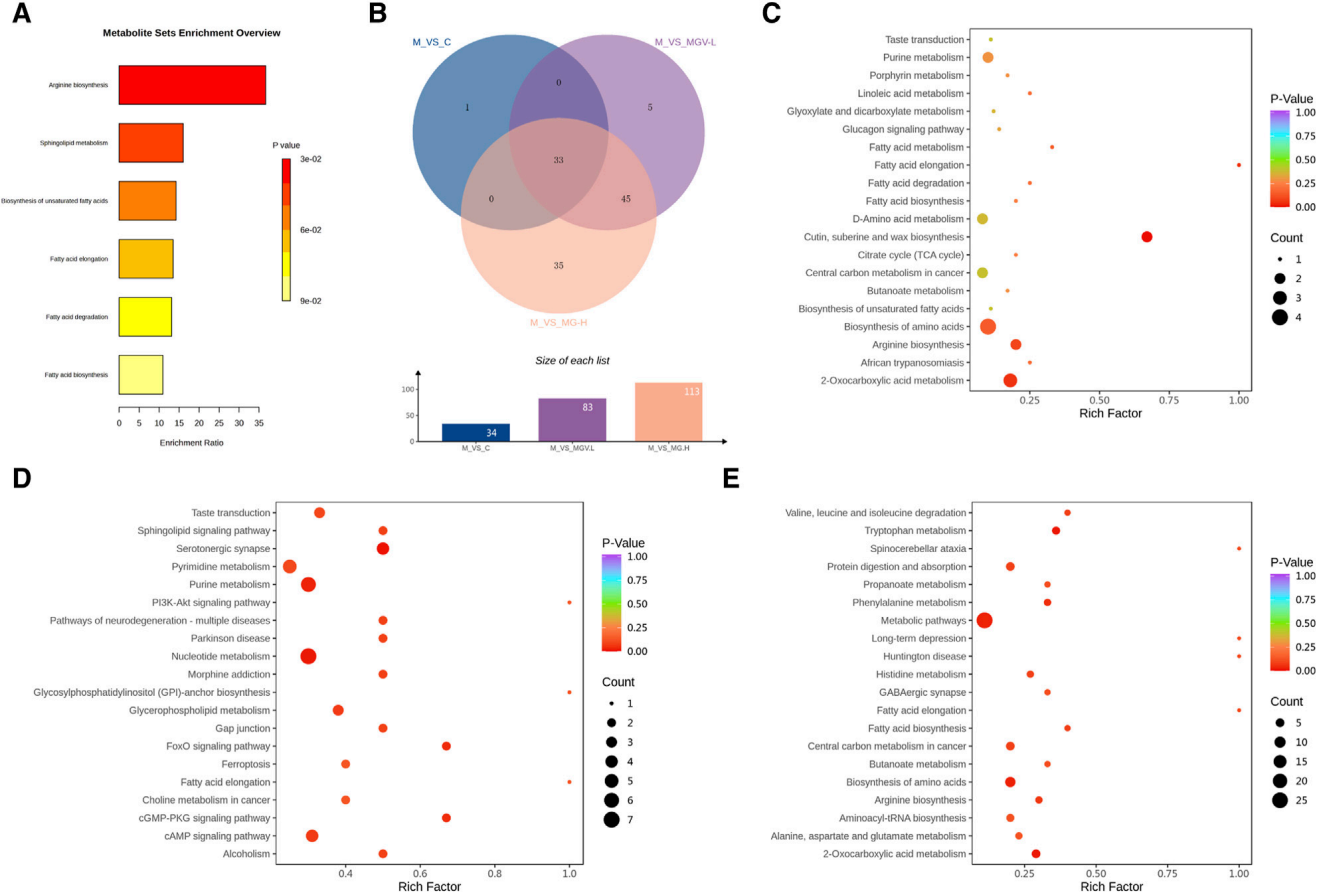
Analysis results of differential metabolic pathways. **(A)** KEGG metabolic pathway enrichment analysis of key differential metabolites. **(B)** Venn diagram showed that 34 differential metabolic pathways between M and C, of which 33 pathways were also enriched in MGV and MG compared with M group. Bubble chart of differential metabolic pathways of model group compared with **(C)** C, **(D)** MGV, **(E)** MG.

Enrichment analyses linked these key metabolites to critical biochemical pathways, such as arginine biosynthesis, sphingolipid metabolism, and fatty acid metabolism, as per the KEGG database (Figure 7). The overarching KEGG enrichment analysis further revealed that mogrosides’ treatment modulated 34 metabolic pathways between the Control and Model groups, with a substantial overlap in pathways affected by both MGV-L and MG-H treatments (Figure 7). This observation suggests that MGV and MG treatments target nearly all metabolic pathways disrupted by MPTP in the disease model, underscoring their potential to rectify PD-associated metabolic dysregulations. Detailed pathway analysis (Figures 7C–E) and (Supplementary Table S4) support the comprehensive impact of mogrosides on metabolic restoration in PD model mice, offering new insights into their therapeutic potential.

## 4 Discussion

The neuroprotective effects of mogrosides, particularly MGV and its metabolite MG, in the context of PD, offer a promising avenue for therapeutic intervention. This study not only corroborates previous findings regarding the neuroprotective properties of mogrosides but also extends our understanding by elucidating their potential to rectify metabolic dysfunctions within the substantia nigra, a critical region affected in PD. The significant reduction in neuronal cell death and restoration of dopaminergic content observed in our models suggest mogrosides may exert a direct neurorestorative effect. This aligns with and enhances the findings from prior research, such as the work by Hanjiang Luo et al. (Luo et al., 2022), which highlighted the neuroprotective capabilities of MGV in both *in vitro* and *in vivo* PD models.

Changes in metabolite levels have a significant impact on neurodegenerative diseases such as PD and AD (Song et al., 2020). The capacity of mogrosides to modulate key metabolic pathways disrupted in PD underscores a potentially groundbreaking approach to treating neurodegenerative diseases. By influencing almost all pathways of metabolic disorder highlighted in our PD model, mogrosides demonstrate a broad spectrum of action that could address the multifactorial etiology of PD. This comprehensive metabolic intervention is particularly compelling given the growing recognition of metabolic dysfunction’s role in PD pathogenesis.

Our discussion on sphingolipid metabolism, pivotal for both cell survival and apoptosis, sheds light on the dual role these metabolites play in determining cell fate. The balance between sphingolipid metabolites, such as ceramide, sphingosine, and sphingosine-1-phosphate, is crucial for autophagy and apoptosis regulation (Shi et al., 2023). These processes are intimately linked to the progressive cell loss characteristic of PD (Motyl et al., 2021). Our findings suggest mogrosides may offer a protective mechanism by stabilizing sphingolipid metabolism, thus safeguarding against autophagy and apoptotic death of neuronal cells. This mechanism could represent a novel therapeutic target, emphasizing the importance of lipid metabolism in neurodegenerative disease progression and treatment.

The study also highlights the normalization of palmitic acid levels following mogrosides treatment, which provides an opportunity to further explore the role of fatty acids in neuroinflammation and PD progression. The regulatory effect of mogrosides on fatty acid distribution suggests a potential neuroprotective mechanism, possibly by attenuating neuroinflammation and oxidative stress (Mi et al., 2023). This is significant considering the exacerbating role of certain fatty acids in inflammatory pathways, contributing to PD’s progression (Shen et al., 2022). Understanding how mogrosides influence fatty acid metabolism could uncover new strategies for mitigating neuroinflammation in PD and other neurodegenerative conditions.

Our examination of amino acid metabolism, particularly the normalization of n-acetyl-l-glutamate, delves into the potential impact of mogrosides on mitochondrial function. Given the central role of mitochondrial dysfunction in PD (Moradi et al., 2023), the observed changes in amino acid profiles are indicative of mogrosides’ contribution to maintaining mitochondrial integrity. This is crucial, as amino acids are not only precursors of neurotransmitters and neuromodulators but also essential for energy metabolism and neuronal communication (Katagiri, 2023). The protection offered by mogrosides on these levels may directly affect neurotransmitter synthesis and release, thereby supporting the function and survival of neurons.

The transition from preclinical findings to clinical applications is the next critical step in evaluating mogrosides’ potential as therapeutic agents for PD. Future research should focus on clinical trials to assess the efficacy, optimal dosing regimen, and long-term safety of mogrosides in human patients. This transition is essential for determining the real-world applicability of our findings and the potential of mogrosides to serve as protective and therapeutic agents against PD. Moreover, the emerging significance of the gut-brain axis in PD underscores the necessity to explore the effects of mogrosides on gut microbiota further (Nishiwaki et al., 2022; Hirayama et al., 2023). Given the positive impact of Siraitia grosvenorii tea on intestinal health (Dong et al., 2021), the therapeutic effects of MGV and its metabolites on PD may extend to improvements in gut microbiome composition and function. This aspect represents a promising research direction, potentially leading to novel therapeutic strategies that leverage the gut-brain axis to combat PD.

## 5 Conclusion

In conclusion, this study enriches the existing body of knowledge by providing novel insights into the neuroprotective mechanisms of MGV and MG, emphasizing their potential to address the complex interplay of factors contributing to PD. The ability of mogrosides to reverse metabolic imbalance, protect mitochondrial function, and possibly influence gut microbiota presents a holistic approach to combating neurodegeneration. As we advance our understanding of PD towards a more comprehensive view of its multifactorial nature, the exploration of natural compounds like mogrosides becomes increasingly vital. The journey from bench to bedside, while challenging, holds the promise of introducing innovative, multi-targeted therapeutic strategies that could significantly improve the quality of life for individuals living with PD.

## Data Availability

The original contributions presented in the study are included in the article/Supplementary Material, further inquiries can be directed to the corresponding authors.

## Supplementary material

SUPPLEMENTARY FIGURE S1 OPLS-DA model validation diagram of Model group compared with **(A)** C, **(B)** MGV, **(C)** MG.

## Abbreviations

Cpu, Caudate putamen; KEGG, Kyoto Encyclopedia of Genes and Genomes; MG, Mogrol; MG-H, High dose of mogrol; MG-L, Low dose of mogrol; MGV, Mogroside V; MGV-H, High dose of mogroside V; MGV-L, Low dose of mogroside V; MPTP, 1-methyl-4-phenyl-1,2,3,6-tetrahydropyridine; OPLS-DA, Orthogonal projections to latent structures discriminant analysis; PCA, Principal component analysis; PD, Parkinson’s disease; SN, Substantia nigra; TH, Tyrosine hydroxylase; VIP, Variable importance in projection.
