# Supplementary material for: Mogroside V and mogrol: unveiling the neuroprotective and metabolic regulatory roles of Siraitia grosvenorii in Parkinson’s disease
Source: Front Pharmacol. 2024 Jul 23;15:1413520. doi: 10.3389/fphar.2024.1413520 (PMC11300226; doi:10.3389/fphar.2024.1413520)
Supplement: Supplementary file 1 [file Table1.pdf]

Supplementary Table 1. Differentially expressed metabolites between Control and Model

Compounds	M1	M2	M3	M4	M5	C1	C2	C3	C4	VIP	p_value	Type
L-Histidine	294810	232560	236900	266970	246800	214540	170510	212980	213680	1.80898	0.015873	down
Citric Acid	3466900	1383200	1748900	1621300	1773300	835670	1121700	1092100	1373100	1.721511	0.015873	down
1-Naphthylacetic Acid	71478	104030	58889	60461	63744	43950	59747	54292	40089	1.649927	0.031746	down
3-Hydroxybutyrate	158560	133610	166890	171040	112650	220570	178190	294520	184550	1.730163	0.015873	up
9,10-DiHOME [(±)9,10-dihydroxy-12Z-octadecenoic acid]	18804	14612	15335	13706	16058	30195	26973	26007	21860	2.146055	0.015873	up
Hexadecanoic Acid(C16:0)	8250400	7964100	7167700	8601600	8034700	9358400	9685800	8638700	8804800	1.796291	0.015873	up
5-Hydroxyhexanoic Acid	18898	21086	16031	17733	21822	26563	23320	25134	21262	1.789498	0.031746	up
Hypoxanthine-9-β-D-Arabinofuranoside	3.1E+08	2.66E+08	2.93E+08	2.77E+08	2.83E+08	2.61E+08	2.55E+08	2.61E+08	2.56E+08	1.862436	0.015873	down
N-Acetylmethionine	63735	55033	63621	59905	68240	46894	36280	46538	40723	2.107461	0.015873	down
ADP-ribose	708340	716440	781660	976030	765930	650680	400820	715120	608630	1.489535	0.031746	down
N-Acetyl-L-alanine	20582000	15730000	19076000	17119000	18798000	16470000	13212000	15543000	12935000	1.776767	0.031746	down
Porphobilinogen	97677	99908	102540	105990	110070	138270	144880	107890	110900	1.650777	0.031746	up
Hexadecanedioic acid	281140	279110	265590	264990	263270	305150	300850	305360	306050	2.20307	0.015873	up
N-acetylornithine	2677400	2281400	2211300	2638500	2573200	1982300	1939700	2098800	1973700	2.018349	0.015873	down
Inosine	3.1E+08	2.66E+08	2.93E+08	2.77E+08	2.83E+08	2.61E+08	2.55E+08	2.61E+08	2.56E+08	1.862436	0.015873	down
N-arachidene glycine	6763.9	6869.4	5953.8	6103.8	6048.8	4777.3	5756	5934	5913.4	1.552049	0.015873	down
12,13-DiHOME	18804	14612	15335	13706	16058	30195	26973	26007	21860	2.146055	0.015873	up
1,11-undecylic acid	1470600	1389000	1520500	1419500	1439900	1609500	1516200	1585500	1613000	1.98863	0.031746	up
3-(pyrazol-1-yl) -L-alanine	474910	357340	395430	405100	362840	321700	272220	354440	317550	1.7736	0.015873	down
2-Methyl-4-pentenoic Acid	292780	285540	317520	303910	331060	409200	332450	416790	318940	1.702302	0.031746	up
N6-Acetyl-L-Lysine	621510	479120	501010	477530	525560	470250	442460	403970	433370	1.723544	0.015873	down
N-Acetyl-L-Glutamic Acid	6319100	5014000	5847300	5586100	5523700	4107400	5170500	4816400	4980600	1.744581	0.031746	down
Β-Nicotinamide Mononucleotide	120780	73572	60561	65263	76071	51881	22221	44557	41038	1.807525	0.015873	down
Dl-2-Aminooctanoic Acid	197160	197620	214990	158320	165890	140080	82618	113720	145040	1.845115	0.015873	down
Kinurenine	76940	57201	46430	55497	42221	35065	32659	46202	40473	1.655787	0.031746	down
Lysopc 15:0	1462000	1306100	1339900	1427100	1446000	1198900	1282700	1313700	1277000	1.807378	0.031746	down
N-Alpha-acetyllysine	621510	479120	501010	477530	525560	470250	442460	403970	433370	1.723544	0.015873	down
4-Aminophenol	25752	15544	18888	24607	19914	30074	34227	33298	37367	2.010893	0.015873	up
Dihydro-D-sphingosine	440570	621860	421470	598870	468480	9	9	9	9	2.352135	0.015078	down
Valyl-leucine	44193	38751	36812	51096	41349	32463	28573	37982	27642	1.804615	0.031746	down

1-O-palmitoyl-2-O-acetyl-sn-glycerol-3-phosphate choline	489330	361520	387550	327480	435130	475060	598340	693800	628040	1.912837	0.031746	up
9-octadecenal	529280	541110	529830	507550	606000	552930	652950	653970	613380	1.656578	0.031746	up
4-tert-butylbenzoic acid	192290	215750	221370	222760	229150	233760	249360	226950	234220	1.544951	0.031746	up
LysoPC 22:5 (2n isomer1)	462840	373690	436910	444740	483140	352870	404650	367890	372780	1.751191	0.031746	down
Carnitine C17:1:DC	715770	633590	684330	729670	616120	500400	582230	564100	496610	2.052815	0.015873	down
Carnitine C18:2-OH	32320	28675	39024	31731	29295	20315	24945	26383	18970	1.959797	0.015873	down
Carnitine C15:DC	601690	481720	548190	590280	550420	407620	480850	420400	354340	2.009231	0.015873	down
Carnitine C14-OH	342060	325440	315380	315050	363190	208890	270180	264700	274140	2.009963	0.015873	down
Carnitine C11:DC	162340	161250	165110	177980	184290	115290	128650	147140	153920	1.85815	0.015873	down
Carnitine C18:1-OH	712720	666810	654790	734460	641620	475130	569490	509060	478010	2.193399	0.015873	down
Carnitine C19	672300	637200	718140	743700	660550	429170	564980	554360	481530	2.073458	0.015873	down

Supplementary Table 2. Differentially expressed metabolites between MGV-L and Model

Compounds	M1	M2	M3	M4	M5	MGV-L1	MGV-L2	MGV-L3	MGV-L4	MGV-L5	VIP	p_value	Type
L-Histidine	294810	232560	236900	266970	246800	174370	220480	220200	211160	149070	1.744625	0.007937	down
L-Tryptophan	232100	200700	282020	253720	250030	137650	205590	204970	195070	124990	1.722175	0.031746	down
L-Cystathionine	210890	219770	216660	222890	223880	171860	169470	204350	202650	98360	1.43961	0.007937	down
N-Acetyl-L-Leucine	51566	43038	46117	57928	42612	33270	41632	39727	45175	36596	1.516229	0.031746	down
N-Propionylglycine	76962	74677	66096	73775	83241	44404	53009	61281	53375	50144	2.080485	0.007937	down
S-(5-Adenosy)-L-Homocysteine	1156400	964270	997200	990080	1020600	851220	968170	928960	978520	867660	1.580438	0.031746	down
Succinic Acid	3909300	3022000	3051200	3372000	5495700	2436900	3225800	2960600	1930800	2946800	1.48895	0.031746	down
Riboflavin	211930	212720	148010	184380	190280	235270	192630	244210	228870	250400	1.646668	0.031746	up
Azelaic Acid	1803100	1965200	1887600	1795100	1745800	2130700	1928300	1968000	1981900	2066900	1.761299	0.015873	up
Methylmalonic Acid	3909300	3022000	3051200	3372000	5495700	2436900	3225800	2960600	1930800	2946800	1.48895	0.031746	down
Palmitoleic Acid(C16:1)	4261700	3971300	3920000	3735200	3943200	2941200	3513300	3299600	3512600	3390900	2.007428	0.007937	down
Hexadecanoic Acid(C16:0)	8250400	7964100	7167700	8601600	8034700	9400100	9946300	8906000	9112300	9748900	2.036057	0.007937	up
Β-Pseudouridine	92652	113240	96343	101330	97720	90037	84378	72401	85761	70287	1.917308	0.007937	down
ADP-ribose	708340	716440	781660	976030	765930	894910	1012400	1440000	876810	1038900	1.657047	0.031746	up
N-Acetylaspartylglutamic acid	30867000	31675000	28997000	31223000	35295000	34339000	40845000	41644000	35924000	36933000	1.872303	0.015873	up
Tetradecanedioic acid	273120	264750	279930	256290	291710	305890	314430	314060	316910	314890	2.107439	0.007937	up
Indoleacetaldehyde	795890	734010	834700	782910	776240	607130	775310	707650	729470	597860	1.633175	0.015873	down
Octadecanamide	598320	585530	576210	580320	584410	649080	631450	644980	682120	652770	2.262019	0.007937	up

2,5-Furandicarboxylicacid	183760	174050	189560	190670	195650	192190	198580	224850	208020	229690	1.794977	0.015873	up
2,4-Hexadienoic acid	1959400	2554000	2160600	2687500	2069300	1644400	1679000	1693000	1659800	1677000	2.02734	0.007937	down
Ricinoleic acid	20488	19051	23474	19732	21573	26401	24942	21218	25220	24600	1.680342	0.031746	up
N-acetylornithine	2677400	2281400	2211300	2638500	2573200	1806400	2066200	2166700	2028700	1467500	1.781703	0.007937	down
Phenylacetic Acid	87563	93009	87075	82566	86614	85638	79625	77205	80239	82093	1.77568	0.015873	down
1,11-undecylic acid	1470600	1389000	1520500	1419500	1439900	1610300	1668500	1646900	1583700	1527800	1.968626	0.007937	up
N,N′-dicyclohexylcarbodiimide	2124700	2581700	2514500	2174700	2413900	2814100	2512000	2692900	3071700	3179300	1.791611	0.031746	up
P-chlorophenylalanine	1779300	1784600	1863700	1848000	1846900	1926700	1891200	2047500	1883300	2025200	1.860769	0.007937	up
Quinolinic acid	10104	11213	10681	10215	10392	8798.8	9736.2	7268.2	9172.5	9174.6	1.866081	0.007937	down
PysoPE 18:2	573960	525430	520880	573020	576660	583780	656230	694070	712100	673190	2.058911	0.007937	up
PysoPE 18:3	30036	29851	32046	29408	27774	29866	35323	36921	41520	35274	1.768454	0.031746	up
L-Glutamic Acid	3.5E+08	2.99E+08	3.16E+08	3.06E+08	3.21E+08	2.73E+08	3.03E+08	2.91E+08	2.81E+08	2.63E+08	1.842342	0.015873	down
Betaine	4081200	3313100	3454500	3969300	3475300	4013300	4964800	4618200	4203400	4009800	1.722727	0.031746	up
Histamine	152690	138510	132540	167500	142660	115410	140310	115150	101650	101460	1.853711	0.031746	down
N-Acetyl-L-Glutamic Acid	6319100	5014000	5847300	5586100	5523700	4316900	5107500	4884700	4682000	4061400	1.894491	0.015873	down
Trimethylamine N-Oxide	36625	25544	40807	23080	25989	66233	70944	63753	88469	70976	2.170921	0.007937	up
Guanosine	57871000	52057000	56097000	62844000	50306000	43765000	53714000	48223000	46853000	40886000	1.719851	0.031746	down
5-Hydroxyindole-3-Acetic Acid	55184	47146	57006	48823	37178	25065	36037	25936	30803	31721	2.074221	0.007937	down
Lysopc 18:2	4499500	4119700	4108600	4711600	3953700	4623000	4707600	5317100	5398200	4985000	1.948888	0.031746	up
N-Acetylglycine	8278300	7173500	8562000	9990200	8011300	11073000	10819000	13614000	11227000	8384200	1.706087	0.031746	up
5'-Deoxy-5'-(Methylthio) Adenosine	27193000	23371000	24618000	27415000	22853000	18555000	20369000	20598000	21174000	16380000	1.945545	0.007937	down
Lysope 18:2 (2N Isomer)	4499500	4119700	4108600	4711600	3953700	4623000	4707600	5317100	5398200	4985000	1.948888	0.031746	up
L-Norleucine	2021300	1889900	2353800	2077200	2211400	1668200	1795300	1903800	1815900	1489600	1.858897	0.015873	down
N-Acetylphenylalanine	102240	91262	109130	113370	80512	56884	78258	74853	84854	56101	1.791621	0.015873	down
2'-Hydroxy-5'-methylacetophenone	244480	338590	256340	275040	273290	316560	361910	304780	419190	552050	1.657233	0.031746	up
Methylcysteine	3213700	2531100	3424400	2605200	3127700	1942800	2585200	1822300	2041900	1645600	2.033447	0.015873	down
Dihydro-D-sphingosine	440570	621860	421470	598870	468480	9	9	9	9	9	2.360886	0.007495	down
2,4-Dihydroxypteridine	54118	34862	67982	38321	36903	32905	25312	26866	37187	28672	1.664017	0.031746	down
1-Single Palm Essence	135400	137010	131980	127700	142090	217560	162640	184230	197770	209520	2.200346	0.007937	up
L-Isoleucine	80781000	64447000	70569000	77518000	79842000	54369000	67013000	62258000	57768000	54529000	1.912898	0.015873	down
9-octadecenal	529280	541110	529830	507550	606000	721320	681370	645050	677330	822590	2.074639	0.007937	up
N-Methyl-α-aminoisobutyric acid	9	25230	34915	15581	21195	38988	37393	43964	59076	37509	1.007189	0.007937	up

Hypotaurocyamine	21843000	21970000	23391000	24705000	21208000	19407000	20919000	20993000	21113000	15507000	1.502024	0.007937	down
5'-deoxy-5'-fluoroadenosine	1728800	1325200	2168000	1869800	1060400	1002900	1701500	974480	1011800	649710	1.532774	0.031746	down
LysoPC 18:2(2n isomer1)	7596000	7422500	7095200	7537200	7305100	8241400	8093400	9683200	9612100	9233000	2.122083	0.007937	up
LysoPC 18:3(2n isomer2)	39410	66092	50892	48097	45548	54771	87088	74906	99852	86665	1.84348	0.015873	up
Carnitine C18:2	137210	139660	93513	100670	142450	54193	99085	77154	88149	54131	1.830359	0.015873	down
Carnitine C14:2-OH	7512.2	8827.7	6160.8	10546	10265	10375	12538	10283	13356	10703	1.660993	0.031746	up
N6-methyladenosine	925340	692660	791660	863540	790260	566130	761150	755060	681400	655660	1.4887	0.031746	down

Supplementary Table 3. Differentially expressed metabolites between MG-H and Model

Compounds	M1	M2	M3	M4	M5	MG-H1	MG-H2	MG-H3	MG-H4	MG-H5	VIP	p_value	Type
Glutathione Reducedform	6201100	7249900	7144500	5054100	6959700	2531000	3139700	5561500	3081300	4886800	1.568766	0.015873	down
3'-Aenylic Acid	309120	206820	236620	236900	269680	143400	158820	202610	147880	115170	1.778166	0.007937	down
Adenosine 5'-Monophosphate	2173800	1303600	1775800	2081500	2119400	1403200	1282500	1239600	1395600	1118000	1.672893	0.031746	down
Succinic Acid	3909300	3022000	3051200	3372000	5495700	2081600	1676300	2537300	2105800	1821200	1.7626	0.007937	down
L-Ascorbate	1618000	1281300	1481900	1795900	1255500	1147300	932120	1286700	1033200	964090	1.686684	0.031746	down
Riboflavin	211930	212720	148010	184380	190280	247220	288820	231960	275980	288700	1.751974	0.007937	up
2-Aminoethanesulfinic Acid	10716	7289.2	7307.3	5988.5	7270.3	13330	10621	11318	11923	11282	1.691011	0.015873	up
Methylmalonic Acid	3909300	3022000	3051200	3372000	5495700	2081600	1676300	2537300	2105800	1821200	1.7626	0.007937	down
O-Phosphorylethanolamine	15654000	13416000	13690000	16186000	13121000	13251000	12807000	13412000	12775000	12975000	1.347647	0.031746	down
Lysope 18:1	26565000	24929000	22672000	24963000	24045000	30837000	35477000	28962000	28799000	29112000	1.740243	0.007937	up
Lysope 18:0	7884000	7308700	7021600	7914300	6899600	10244000	8463000	9037000	8097200	9430500	1.639165	0.007937	up
Lysope 16:0	17689000	15504000	15440000	16258000	16536000	19094000	21477000	19612000	17457000	19460000	1.683997	0.015873	up
Lysope 14:0	67192	56159	60286	60174	63184	66065	89616	70925	75002	67533	1.456202	0.015873	up
Hexadecanoic Acid(C16:0)	8250400	7964100	7167700	8601600	8034700	8887400	9581600	8774600	8787200	8947200	1.572184	0.007937	up
DHA [4Z,7Z,10Z,13Z,16Z,19Z-docosahexaenoic acid]	655900	611940	605580	628820	621530	734800	639470	661830	672010	689960	1.479365	0.015873	up
2'-Deoxycytidine-5'-Monophosphate	147990	146020	240050	262870	211720	87600	102060	135500	120280	118130	1.686831	0.007937	down
Aminomalonic Acid	134280	117910	90855	120610	202310	77791	42738	86644	72186	64361	1.637583	0.007937	down
8,15-Dihete	613690	572860	595810	537020	613330	737690	738400	709540	715910	643460	1.811667	0.007937	up
N-Acetylmethionine	63735	55033	63621	59905	68240	46480	32701	59685	45512	48729	1.505667	0.015873	down
Xanthosine	1713200	1439900	1390300	1420300	1458300	2167000	2417000	1702600	1683200	2720800	1.588621	0.031746	up
N-Acetyl-L-alanine	20582000	15730000	19076000	17119000	18798000	15893000	14187000	14269000	10995000	12476000	1.707993	0.015873	down
Glycerol 3-phosphate	6387800	4656600	4342200	6580300	8179400	4220400	3009800	4654100	2845700	2816800	1.626316	0.015873	down

deoxyguanosine 5'-monophosphate(dGMP)	2173800	1303600	1775800	2081500	2119400	1403200	1282500	1239600	1395600	1118000	1.672893	0.031746	down
Tetradecanedioic acid	273120	264750	279930	256290	291710	299680	295920	312350	313280	304490	1.753819	0.007937	up
Octadecanamide	598320	585530	576210	580320	584410	611230	648760	628400	625890	659490	1.84104	0.007937	up
(±)5-HETE [(±)5-hydroxy-6E,8Z,11Z,14Z-eicosatetraenoic acid]	20378	34254	17285	12646	16868	41074	41055	38556	33193	32586	1.699983	0.031746	up
(±)9-HETE [(±)-9-hydroxy-5Z,7E,11Z,14Z-eicosatetraenoic acid]	20378	34254	17285	12646	16868	41074	41055	38556	33193	32586	1.699983	0.031746	up
PGJ2 [11-oxo-15S-hydroxy-prosta-5Z,9,13E-trien-1-oic acid]	60027	51781	58845	39553	46652	83465	60264	54625	65554	100670	1.371533	0.031746	up
3-Hydroxyglutaric acid	166790	151480	125210	173870	157930	112950	102260	127920	126150	107270	1.71952	0.031746	down
3-Ureidopropionate	408990	298240	310020	362780	290300	268480	237560	287540	226820	281200	1.575927	0.007937	down
3-hydroxyphenylacetic acid	53536	37713	39212	73345	63535	112560	83777	76747	81951	59595	1.37531	0.031746	up
Homo-Gamma-Linolenic Acid	4073000	3316900	3109000	3233100	3697400	4209000	4105600	3814900	4365600	4071000	1.514323	0.031746	up
N-arachidene glycine	6763.9	6869.4	5953.8	6103.8	6048.8	7104.5	7042.3	7568.3	7401	6995	1.66954	0.007937	up
(4E,7E,10Z,13E,16E,19E) -docosa-4,7,10,13,16,19-hexanoic acid	655900	611940	605580	628820	621530	734800	639470	661830	672010	689960	1.479365	0.015873	up
UDP-D-galactose	6238100	6541100	6199400	10779000	5772700	4486500	4570800	5938600	5285500	5656800	1.33734	0.015873	down
1,11-undecylic acid	1470600	1389000	1520500	1419500	1439900	1526900	1528500	1632700	1482800	1607800	1.477543	0.015873	up
L-arabinonic acid-1,4-lactone	166790	151480	125210	173870	157930	112950	102260	127920	126150	107270	1.71952	0.031746	down
N,N′-dicyclohexylcarbodiimide	2124700	2581700	2514500	2174700	2413900	2900500	3148000	2573500	2721900	2683700	1.614715	0.015873	up
5-O-(1-carboxyvinyl) -3-phosphate	738910	446250	594860	608160	699470	357800	347690	351540	280490	213340	1.857565	0.007937	down
PysoPE 22:4(2n isomer1)	1945500	1685900	1778900	2011100	1829900	2112500	2658700	2170300	1935500	2306700	1.47189	0.031746	up
PysoPE 22:4	1945500	1685900	1778900	2011100	1829900	2112500	2658700	2170300	1935500	2306700	1.47189	0.031746	up
PysoPE 22:5(2n isomer3)	217570	173840	192690	222550	166410	242350	336220	309650	216760	314280	1.586261	0.031746	up
PysoPE 22:5(2n isomer2)	217570	173840	192690	222550	166410	242350	336220	309650	216760	314280	1.586261	0.031746	up
PysoPE 22:5(2n isomer1)	217570	173840	192690	222550	166410	242350	336220	309650	216760	314280	1.586261	0.031746	up
PysoPE 20:3(2n isomer1)	438730	391320	417840	466050	391520	497710	636470	524380	496280	539700	1.696551	0.007937	up
PysoPE 20:3	438730	391320	417840	466050	391520	497710	636470	524380	496280	539700	1.696551	0.007937	up
PysoPE 20:5(2n isomer1)	44106	44876	50690	47845	41166	51112	63579	56586	48368	65541	1.530317	0.015873	up
PysoPE 20:5	44106	44876	50690	47845	41166	51112	63579	56586	48368	65541	1.530317	0.015873	up
PysoPE 18:0(2n isomer)	7884000	7308700	7021600	7914300	6899600	10244000	8463000	9037000	8097200	9430500	1.639165	0.007937	up
PysoPE 18:2	573960	525430	520880	573020	576660	700900	819230	627420	672900	687980	1.757009	0.007937	up
PysoPE 18:3(2n isomer)	31271	26058	27508	32156	24835	35210	42592	36956	35479	35243	1.684273	0.007937	up
PysoPE 18:3	30036	29851	32046	29408	27774	38497	37622	33423	36267	35507	1.849475	0.007937	up
PysoPE 16:0(2n isomer)	17689000	15504000	15440000	16258000	16536000	19094000	21477000	19612000	17457000	19460000	1.683997	0.015873	up
PysoPE 16:1	1313100	1126300	1063300	1076700	1086400	1555700	1657600	1473300	1337200	1290700	1.677974	0.015873	up

N-Carbamoyl-L-aspartate	2755800	2381500	2280800	2823500	2057400	2032200	1426800	2378200	1596800	1666700	1.523322	0.031746	down
L-Glutamic Acid	3.5E+08	2.99E+08	3.16E+08	3.06E+08	3.21E+08	2.58E+08	2.56E+08	2.94E+08	2.9E+08	2.66E+08	1.700248	0.007937	down
N-Acetyl-L-Glutamic Acid	6319100	5014000	5847300	5586100	5523700	5474900	4739100	4342700	4817700	4984800	1.50259	0.015873	down
Serotonin	200950	113270	171370	131370	154300	44935	61938	76921	62306	52971	1.93366	0.007937	down
Trimethylamine N-Oxide	36625	25544	40807	23080	25989	45349	53228	47074	73940	80284	1.716727	0.007937	up
1,4-Dihydro-1-Methyl-4-Oxo-3-Pyridinecarboxamide	2360200	2444500	2684500	2918000	2408400	1997200	2081500	2163400	2103500	2099000	1.794345	0.007937	down
Adenosine	2.22E+08	1.78E+08	2.6E+08	2.34E+08	1.48E+08	1.06E+08	1.24E+08	1.57E+08	1.14E+08	1.35E+08	1.713186	0.015873	down
Cytidine-5-Monophosphate	4084900	2512900	2366000	2875500	2843500	2021300	1893200	2470900	2363800	1910000	1.503826	0.015873	down
Guanosine	57871000	52057000	56097000	62844000	50306000	41355000	44620000	49909000	42160000	47516000	1.725998	0.007937	down
5-Hydroxyindole-3-Acetic Acid	55184	47146	57006	48823	37178	29035	29007	40201	33754	32973	1.719491	0.015873	down
Lysopc 18:2	4499500	4119700	4108600	4711600	3953700	4887600	6541800	4297500	4958300	5723400	1.37734	0.031746	up
Lysopc 20:1	6644700	5297300	5407200	6521100	6974000	7755400	9234300	7360200	6954800	7744200	1.515422	0.015873	up
N6-Succinyl Adenosine	9583300	8194000	8532000	8258100	6946700	7177100	5886100	5992000	5035100	5090900	1.766723	0.015873	down
L-Homoarginine	35262	29356	58856	65543	38715	80451	100480	45414	67201	117860	1.380844	0.031746	up
5'-Deoxy-5'-(Methylthio) Adenosine	27193000	23371000	24618000	27415000	22853000	22806000	22859000	21714000	18822000	20426000	1.588898	0.015873	down
Sn-Glycero-3-Phosphocholine	31159000	36614000	47391000	53596000	34852000	19033000	15731000	32641000	26873000	17483000	1.642448	0.015873	down
Lysopc 15:0	1462000	1306100	1339900	1427100	1446000	1592100	1961100	1446700	1485800	1519800	1.255451	0.015873	up
Lysope 18:2 (2N Isomer)	4499500	4119700	4108600	4711600	3953700	4887600	6541800	4297500	4958300	5723400	1.37734	0.031746	up
Isobutyryl carnitine	967080	1215000	1097400	1532400	1221900	652680	968410	958730	787560	1010300	1.453878	0.031746	down
LysoPE(16:1(9Z)/0:0)	56618	49477	44137	59151	44799	60064	73938	56688	63991	61685	1.466853	0.015873	up
Dihydro-D-sphingosine	440570	621860	421470	598870	468480	5126.6	31067	41499	21953	28378	1.944568	0.007937	down
N-Methyl-L-Glutamate	1255600	1145900	1526300	1645900	1437900	895860	942510	943660	1260800	912870	1.670195	0.031746	down
Allopurinol	86891000	88186000	87700000	94579000	83981000	81349000	80028000	87147000	75903000	82228000	1.484143	0.031746	down
PGD2 [9α,15S-dihydroxy-11-oxo-prosta-5Z,13E-dien-1-oic acid]	93194	77194	99346	72825	72080	121210	101020	102210	91159	136510	1.462589	0.031746	up
1-Single Palm Essence	135400	137010	131980	127700	142090	154430	219950	171760	164460	172690	1.728007	0.007937	up
PE (18: 1 (9Z) / 0: 0)	32869000	24669000	25796000	31270000	28552000	37597000	42344000	37501000	33462000	34868000	1.609274	0.007937	up
1-O-palmitoyl-2-O-acetyl-sn-glycerol-3-phosphate choline	489330	361520	387550	327480	435130	616480	992370	519280	496660	498770	1.437829	0.007937	up
9-octadecenal	529280	541110	529830	507550	606000	647720	670740	659170	561380	689050	1.655716	0.015873	up
Hypotaurocyamine	21843000	21970000	23391000	24705000	21208000	17949000	18873000	20631000	17818000	20178000	1.729976	0.007937	down
5'-deoxy-5'-fluoroadenosine	1728800	1325200	2168000	1869800	1060400	636040	789710	1164400	798900	1005400	1.664646	0.015873	down
N-acetylpyrrolidine	2369300	2279300	2291700	2246200	2354900	2148700	2089700	2286600	2138500	2034900	1.632457	0.031746	down
LysoPC 22:5 (2n isomer3)	468170	401800	379110	484850	443000	527920	641830	605540	449370	571350	1.506298	0.031746	up

LysoPC 22:5 (2n isomer2)	468170	401800	379110	484850	443000	527920	641830	605540	449370	571350	1.506298	0.031746	up
LysoPC 18:0(2n isomer)	61759000	56313000	56773000	64568000	63762000	66782000	80594000	70066000	67020000	71532000	1.595561	0.007937	up
LysoPC 18:2(2n isomer1)	7596000	7422500	7095200	7537200	7305100	9193000	10909000	7841600	8734400	9756300	1.684487	0.007937	up
LysoPC 16:0(2n isomer)	3.87E+08	3.37E+08	3.75E+08	3.62E+08	3.66E+08	3.97E+08	4.29E+08	3.67E+08	3.88E+08	3.97E+08	1.275717	0.031746	up
LysoPC 16:1(2n isomer)	1842800	1500700	1736600	1633100	1447300	1891900	2264800	1881700	1659600	1846200	1.253814	0.031746	up
Carnitine C22:2	109600	99061	91947	93215	95792	132190	143780	117160	113800	126030	1.771478	0.007937	up
Carnitine C4:DC	1522900	1267700	1278100	1494300	1357400	1350700	1252400	1193900	1238400	1161100	1.398719	0.031746	down
Carnitine C5:0	433940	523150	452310	631420	516110	375780	509010	307080	422790	355130	1.342265	0.031746	down
Carnitine C5:1	91744	83598	91670	103060	93668	86292	80552	73371	83478	51259	1.330345	0.015873	down
1-Methyl-6-oxo-1,6-dihydropyridine-3-carboxamide	252630	222650	267290	260170	218960	184590	166860	205140	188990	171660	1.852583	0.007937	down
Quinoline-4-carboxylic acid	139230	116620	124170	92599	117210	54975	75864	114060	94478	74219	1.416426	0.031746	down
Carnitine C10:0 Isomer1	26854	39358	30739	32099	33376	20234	26242	30377	24438	21138	1.453689	0.015873	down

Supplementary Table 4. Kegg pathway of differentially expressed metabolites

No.	M_VS_C	M_VS_MGV-L	M_VS_MG-H
1	Cutin, suberine and wax biosynthesis	Tryptophan metabolism	Serotonergic synapse
2	2-Oxocarboxylic acid metabolism	2-Oxocarboxylic acid metabolism	Nucleotide metabolism
3	Fatty acid elongation	Metabolic pathways	Purine metabolism
4	Arginine biosynthesis	Biosynthesis of amino acids	cGMP-PKG signaling pathway
5	Biosynthesis of amino acids	Phenylalanine metabolism	FoxO signaling pathway
6	Fatty acid metabolism	Arginine biosynthesis	Glycerophospholipid metabolism
7	Fatty acid degradation	Valine, leucine and isoleucine degradation	cAMP signaling pathway
8	African trypanosomiasis	Fatty acid biosynthesis	Pathways of neurodegeneration - multiple diseases
9	Linoleic acid metabolism	Histidine metabolism	Gap junction
10	Citrate cycle (TCA cycle)	Protein digestion and absorption	Parkinson disease
11	Fatty acid biosynthesis	Central carbon metabolism in cancer	Alcoholism
12	Butanoate metabolism	Spinocerebellar ataxia	Sphingolipid signaling pathway
13	Porphyrin metabolism	Huntington disease	Morphine addiction
14	Purine metabolism	Fatty acid elongation	Taste transduction
15	Glucagon signaling pathway	Long-term depression	Pyrimidine metabolism
16	Glyoxylate and dicarboxylate metabolism	GABAergic synapse	Choline metabolism in cancer
17	D-Amino acid metabolism	Propanoate metabolism	Ferroptosis

18	Central carbon metabolism in cancer	Butanoate metabolism	Spinocerebellar ataxia
19	Taste transduction	Aminoacyl-tRNA biosynthesis	Ether lipid metabolism
20	Biosynthesis of unsaturated fatty acids	Alanine, aspartate and glutamate metabolism	Long-term depression
21	Lysine degradation	Synaptic vesicle cycle	Huntington disease
22	Nicotinate and nicotinamide metabolism	Cysteine and methionine metabolism	Fatty acid elongation
23	Metabolic pathways	Serotonergic synapse	PI3K-Akt signaling pathway
24	Tryptophan metabolism	Glyoxylate and dicarboxylate metabolism	Glycosylphosphatidylinositol (GPI)-anchor biosynthesis
25	Histidine metabolism	Asthma	Glutathione metabolism
26	cAMP signaling pathway	Circadian entrainment	Metabolic pathways
27	Alanine, aspartate and glutamate metabolism	Long-term potentiation	Regulation of lipolysis in adipocytes
28	Biosynthesis of cofactors	Shigellosis	Butanoate metabolism
29	beta-Alanine metabolism	Nitrogen metabolism	Propanoate metabolism
30	Carbon metabolism	Amyotrophic lateral sclerosis	Renin secretion
31	Aminoacyl-tRNA biosynthesis	Glycine, serine and threonine metabolism	GABAergic synapse
32	ABC transporters	Nicotinate and nicotinamide metabolism	Neuroactive ligand-receptor interaction
33	Nucleotide metabolism	C5-Branched dibasic acid metabolism	Synaptic vesicle cycle
34	Protein digestion and absorption	Nicotine addiction	Alanine, aspartate and glutamate metabolism
35		Retrograde endocannabinoid signaling	Long-term potentiation
36		Gastric acid secretion	Circadian entrainment
37		Cutin, suberine and wax biosynthesis	Olfactory transduction
38		Axon regeneration	Asthma
39		Fc epsilon RI signaling pathway	Cortisol synthesis and secretion
40		Amphetamine addiction	Cushing syndrome
41		Pyruvate metabolism	Antifolate resistance
42		Oxidative phosphorylation	Amyotrophic lateral sclerosis
43		Glutamatergic synapse	Nitrogen metabolism
44		Fatty acid metabolism	Arachidonic acid metabolism
45		FoxO signaling pathway	Taurine and hypotaurine metabolism
46		Cocaine addiction	Glyoxylate and dicarboxylate metabolism
47		Riboflavin metabolism	Biosynthesis of unsaturated fatty acids
48		ABC transporters	Phenylalanine metabolism
49		Neuroactive ligand-receptor interaction	Vitamin digestion and absorption

50		Fatty acid degradation	AMPK signaling pathway
51		Alcoholism	Sphingolipid metabolism
52		African trypanosomiasis	Axon regeneration
53		Chemical carcinogenesis - reactive oxygen species	mTOR signaling pathway
54		Gap junction	Nicotine addiction
55		Pathways of neurodegeneration - multiple diseases	Cutin, suberine and wax biosynthesis
56		Proximal tubule bicarbonate reclamation	Vascular smooth muscle contraction
57		Phospholipase D signaling pathway	Retrograde endocannabinoid signaling
58		Neomycin, kanamycin and gentamicin biosynthesis	Pyruvate metabolism
59		Valine, leucine and isoleucine biosynthesis	Glycerolipid metabolism
60		Biosynthesis of cofactors	Aldosterone synthesis and secretion
61		Citrate cycle (TCA cycle)	Riboflavin metabolism
62		Inflammatory mediator regulation of TRP channels	Cocaine addiction
63		Ferroptosis	Amphetamine addiction
64		beta-Alanine metabolism	Parathyroid hormone synthesis, secretion and action
65		D-Amino acid metabolism	C5-Branched dibasic acid metabolism
66		Mineral absorption	Fatty acid metabolism
67		Carbon metabolism	Oxidative phosphorylation
68		Porphyrin metabolism	Longevity regulating pathway
69		Phenylalanine, tyrosine and tryptophan biosynthesis	Glutamatergic synapse
70		Glucagon signaling pathway	Fc epsilon RI signaling pathway
71		Sulfur metabolism	ABC transporters
72		Taurine and hypotaurine metabolism	Nicotinate and nicotinamide metabolism
73		Pyrimidine metabolism	Arginine biosynthesis
74		Purine metabolism	HIF-1 signaling pathway
75		Biosynthesis of unsaturated fatty acids	African trypanosomiasis
76		Vitamin digestion and absorption	Caffeine metabolism
77		Taste transduction	Proximal tubule bicarbonate reclamation
78		Lysine degradation	Phospholipase D signaling pathway
79		Glutathione metabolism	Chemical carcinogenesis - receptor activation
80		cAMP signaling pathway	Chemical carcinogenesis - reactive oxygen species
81		Tyrosine metabolism	Fatty acid degradation

82		Arginine and proline metabolism	Neomycin, kanamycin and gentamicin biosynthesis
83		Nucleotide metabolism	Tryptophan metabolism
84			Fatty acid biosynthesis
85			Inflammatory mediator regulation of TRP channels
86			Diabetic cardiomyopathy
87			Valine, leucine and isoleucine degradation
88			Citrate cycle (TCA cycle)
89			Tyrosine metabolism
90			Phenylalanine, tyrosine and tryptophan biosynthesis
91			Porphyrin metabolism
92			Ascorbate and aldarate metabolism
93			Cysteine and methionine metabolism
94			Bile secretion
95			Carbon metabolism
96			Glucagon signaling pathway
97			Thyroid hormone synthesis
98			Sulfur metabolism
99			2-Oxocarboxylic acid metabolism
100			Biosynthesis of cofactors
101			Pantothenate and CoA biosynthesis
102			Galactose metabolism
103			Lysine degradation
104			Biosynthesis of nucleotide sugars
105			Histidine metabolism
106			Amino sugar and nucleotide sugar metabolism
107			D-Amino acid metabolism
108			beta-Alanine metabolism
109			Central carbon metabolism in cancer
110			Arginine and proline metabolism
111			Biosynthesis of amino acids
112			Aminoacyl-tRNA biosynthesis
113			Protein digestion and absorption
