# Supplementary figures and images for: Mogroside V and mogrol: unveiling the neuroprotective and metabolic regulatory roles of Siraitia grosvenorii in Parkinson’s disease
Source: Front Pharmacol. 2024 Jul 23;15:1413520. doi: 10.3389/fphar.2024.1413520 (PMC11300226; doi:10.3389/fphar.2024.1413520)

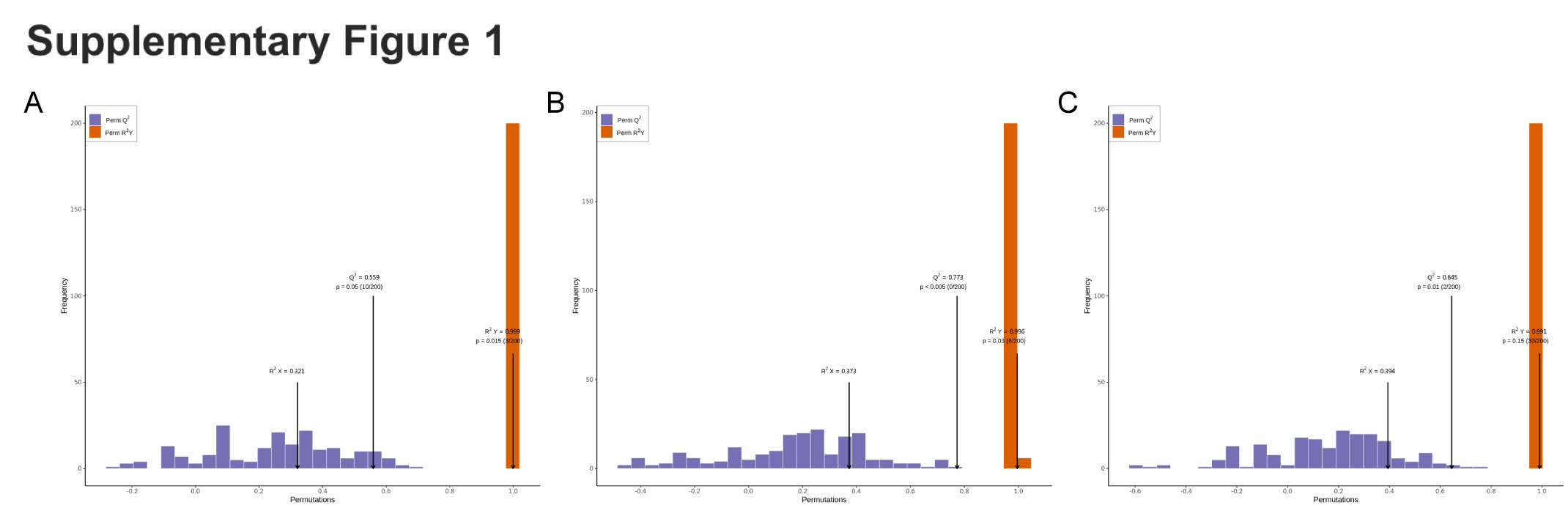

Supplement: Supplementary file 2 [file Image1.TIF]
