# Supplementary material for: Mogroside V and mogrol: unveiling the neuroprotective and metabolic regulatory roles of Siraitia grosvenorii in Parkinson’s disease
Source: Front Pharmacol. 2024 Jul 23;15:1413520. doi: 10.3389/fphar.2024.1413520 (PMC11300226; doi:10.3389/fphar.2024.1413520)

Supplementary Material

**Supplementary Figure 1.** OPLS-DA model validation diagram of Model group compared with (A) C, (B) MGV, (C) MG.


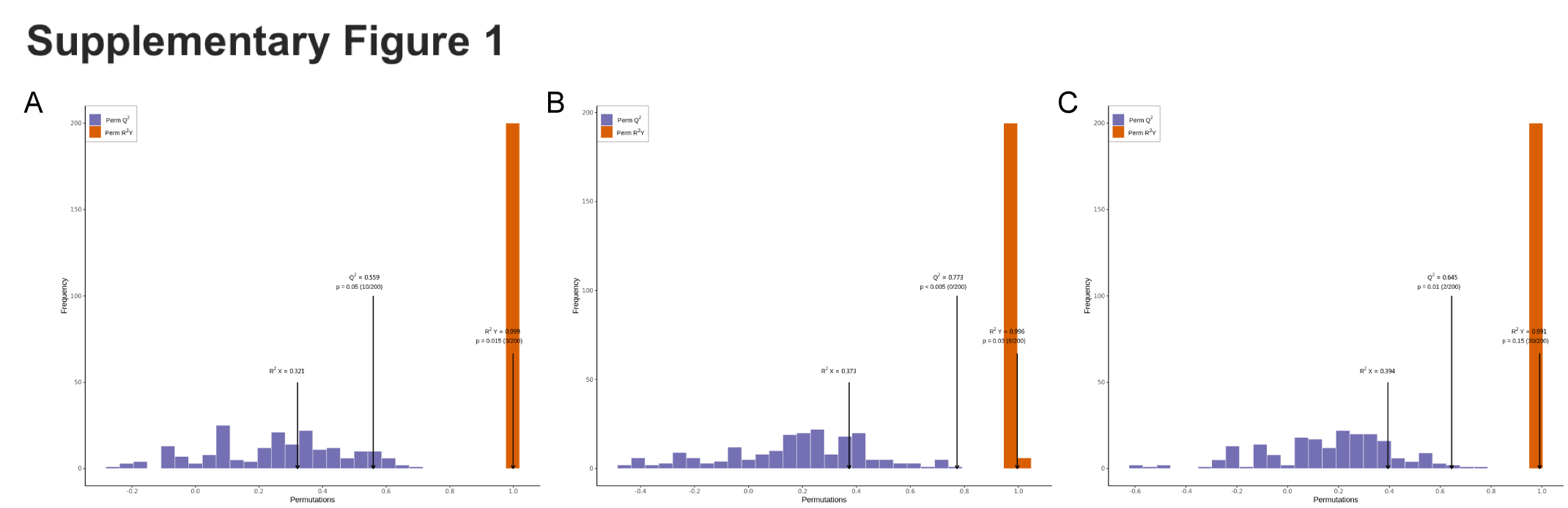

Supplement: Supplementary file 3 [file DataSheet1.docx]
